# The BinaxNOW pneumococcal antigen test: An adjunct for diagnosis of pneumococcal bacteraemia

**DOI:** 10.4102/sajid.v36i1.244

**Published:** 2021-04-15

**Authors:** Hafsah D. Tootla, Colleen Bamford, Chad M. Centner, Clinton Moodley

**Affiliations:** 1 Division of Medical Microbiology, Faculty of Health Sciences, University of Cape Town, Cape Town, South Africa; 2 National Health Laboratory Service, Microbiology, Groote Schuur Hospital, Cape Town, South Africa

**Keywords:** *Streptococcus pneumoniae*, bacteraemia, BinaxNOW, antigen, pneumococcal diagnosis, test

## Abstract

**Background:**

Culture remains the diagnostic standard for *Streptococcus pneumoniae* bacteraemia but is limited by time to identification, prior antibiotics and bacterial autolysis. Culture-independent methods for detecting *S. pneumoniae* include PCR and antigen tests. We evaluated an antigen test on blood culture broth for the rapid detection of *S. pneumoniae* bacteraemia.

**Method:**

We collected 212 signal-positive blood cultures, with gram-positive cocci in pairs, chains or with uncertain morphology. The BinaxNOW *S. pneumoniae* urinary antigen test, Gram stain, culture and *lytA* PCR were performed on all samples. Diagnostic accuracy of the antigen test and Gram stain with gram-positive cocci in pairs were compared with culture, polymerase chain reaction (PCR) and the composite of culture and PCR.

**Results:**

*Streptococcus pneumoniae* was isolated in 26% of samples, 66% cultured other gram-positive organisms and 8% of samples had no growth. Sensitivity and negative predictive values of the antigen test were 100%, specificity and positive predictive values were 87% – 88% and 76% – 81%, but increased to 93% – 96% and 96% – 98% when applied to subsets with gram-positive cocci in pairs, or history compatible with respiratory illness or meningitis. Sensitivity (69% – 75%) and specificity (81%) of Gram stain (gram-positive cocci in pairs) were lower than the antigen test even when applied to the same subsets.

**Conclusion:**

Accurate and rapid diagnosis of *S. pneumoniae* bacteraemia is challenging. Specificity of this antigen test is limited by cross-reactivity with other gram-positive organisms, but could be improved if Gram stain morphology and clinical history are available. The antigen test is a useful adjunct for rapid diagnosis of *S. pneumoniae* bacteraemia.

## Introduction

Invasive pneumococcal disease (IPD) remains a cause of significant morbidity and mortality worldwide despite the introduction of pneumococcal conjugate vaccination programmes.^1,2,3^

The diagnosis of IPD from sterile sites traditionally relies on microscopy indicating gram-positive cocci in pairs and culture of the organism.^4^ Identification of *Streptococcus pneumoniae* from culture is confirmed by phenotypic methods, such as susceptibility to optochin (ethylhydrocupreine) and bile solubility. *Streptococcus pneumoniae* is a fastidious organism, easily inhibited by prior antibiotic exposure and prone to autolysis under laboratory conditions.

Culture is also slow, taking 24 – 48 h for definitive identification.^4,5,6^ Fortunately, high-level penicillin resistance, defined as minimum inhibitory concentration (MIC) ≥ 2 µg/mL, is still relatively rare (~7%) in our setting.^1^ Rapid testing would allow for rapid de-escalation of empiric therapy in non-meningitis infections, reducing the selection of resistance.

Alternative culture-independent methods for the diagnosis of IPD include PCR and antigen tests. Polymerase chain reaction (PCR) is more sensitive, less influenced by prior antibiotic exposure, and rapid, but is limited by cost, accessibility and variable diagnostic accuracy depending on the gene target used. Most of the currently used gene targets (*ply, lytA, psaA, sodA* and *pbp*) demonstrated reduced specificity, primarily because of cross-reactivity with other closely related species.^4,7,8,9^

The BinaxNOW *S. pneumoniae* Antigen Card test (Alere) is a lateral flow immunochromatographic test (ICT), which detects C-polysaccharide cell wall protein, common to all *S. pneumoniae* serotypes.^6,8,10,11^ It has been validated for use on urine to assist with the diagnosis of community-acquired pneumonia (CAP), although cross-reactivity with other streptococcal species and *S. pneumoniae* colonisation in the upper respiratory tract have limited its utility. The test can also be used on specimens from sterile sites such as cerebrospinal fluid for the rapid diagnosis of pneumococcal meningitis. A few studies have explored its use on incubated signal-positive blood cultures, demonstrating its potential for rapid identification of *S. pneumoniae* bacteraemia, especially when culture does not yield growth of an organism. However, differences in reference methods and inclusion criteria have hindered the comparisons of diagnostic accuracy.^5,6,12,13^

We compared the BinaxNOW *S. pneumoniae* Antigen Card ICT with a rigorous reference standard, incorporating both culture and *lytA* PCR, to determine if this test could rapidly identify *S. pneumoniae* from selected signal-positive blood cultures.

## Methods

### Study setting and design

A prospective study was carried out at the National Health Laboratory Service, Groote Schuur Microbiology Laboratory, Cape Town, South Africa, between 01 June 2017 and 31 March 2018. The laboratory provides diagnostic microbiology services to ~1.85 million people from predominantly low socio-economic backgrounds. We receive approximately 40 000 blood cultures annually and use the BacT/ALERT automated blood culture system (BioMérieux, Marcy-l’Etoile, France) for routine testing.

A convenience sampling method was employed, where non-consecutive signal-positive blood culture bottles with microscopy suggestive of *S. pneumoniae*, namely, gram-positive cocci in pairs or chains, or with gram positive cocci in uncertain morphological arrangement, were selected by a single researcher. Samples were only collected during periods of researcher availability in the laboratory. The presence of brown-coloured broth suggestive of autolysis was subjectively noted. Antigen testing was performed within 6 h of Gram staining. Deoxyribonucleic acid extraction and PCR were batched and performed on all samples by the single researcher who was blinded to the results of the other diagnostic tests and clinical presentation, until all tests were completed. Basic clinical and epidemiological information were collected by the attending microbiology trainee through telephonic consultation with the treating doctor and notes were made from the clinical liaison.

### Culture

Blood culture broth from signal-positive bottles was aspirated and Gram staining was performed.

Samples exhibiting gram-positive cocci in pairs, chains or gram-positive cocci of uncertain morphological arrangement were inoculated onto 2% horse blood agar and 5% sheep blood agar, the latter with a 5 µg optochin disc placed in the main inoculating zone, as per routine laboratory standard operating procedures (SOPs). Both plates were incubated in 5% CO_2_ at 37 °C and inspected after 24 and 48 h. *Streptococcus pneumoniae* was identified by the presence of alpha-haemolytic streptococci with a zone of inhibition around the optochin disk ≥ 14 mm diameter.^14^ Alpha-haemolytic streptococci with zones of inhibition < 14 mm diameter were further identified using the Vitek 2 GP-ID card (BioMérieux, Marcy-l’Etoile, France), according to the manufacturer’s instructions.

### BinaxNOW *Streptococcus pneumoniae* antigen card test

Swabs provided by the manufacturer were submerged into 1 mL aliquots of blood culture broth and inserted into the test card. Test reagent was added and the card sealed. Results were read after 15 min and interpreted according to the package insert.^15^ Faint reactive lines were recorded as weak positive results.

### Deoxyribonucleic acid extraction

Deoxyribonucleic acid extraction of blood culture broth was performed using the QIAsymphony SP automated extraction instrument with the QIAsymphony Virus/Bacteria mini kit, according to the manufacturer’s instructions (Qiagen, Hilden, Germany). Deoxyribonucleic acid extracted from 200 µL of blood culture broth was eluted into 60 µL of elution buffer and stored at –70 °C.

### Real-time *lytA* polymerase chain reaction

Polymerase chain reaction assays were batched and performed using the Rotor-Gene 6000 (Corbett Research) analyser. Each 25 µL reaction contained 5 µL of extracted DNA (at a 1:10 dilution to reduce the effect of co-purified inhibitors from blood culture broth), 200 nM each of the forward and reverse primer and probe (Table 1-A1),^16^ 12.5 µL of LightCycler^®^ 480 Probes Master (Roche Life Science) master mix and 6 µL of PCR grade water. A no template control and *S. pneumoniae* positive DNA control (*S. pneumoniae* ATCC 49619) were included in every run. Deoxyribonucleic acid was amplified using the following cycling conditions: 95 °C for 5 min, then 40 cycles at 95 °C for 10 s and 60 °C for 1 min with fluorescence detection.

Results were analysed using the Rotor-Gene 6000 Series Software 1.7. Both ICT and PCR were repeated on all discordant samples, with no changes to the initial results noted.

A cycle threshold (CT) cut-off value of ≤ 22 was used to determine PCR positivity. This cut-off value was experimentally determined by receiver operating characteristic (ROC) curve analysis (Figure 1-A1) and inspection of CT-value distributions of signal-positive samples (Figure 2-A1). For ROC curve analysis we considered *S. pneumoniae* culture-positive samples as true positives, and those with organisms other than *S. pneumoniae* as true negatives. Receiver operating characteristic curve analysis suggested that the optimal CT cut-off value for determining a positive PCR result was between 17 and 22 (area under the curve [AUC] 0.99) and the upper value of CT ≤ 22 was selected.

### Sample size

Using the following parameters – prevalence of *S. pneumoniae* from blood cultures in our laboratory with gram-positive cocci in pairs/chains or cocci with uncertain morphological arrangement = 20%, hypothesised sensitivity and specificity of ICT using signal-positive blood culture broth = 99% and 82%, respectively,^5,6,12,13^ desired precision = 95% and desired confidence interval (CI) = 90% – we estimated the required sample size to be ≥ 201.^17,18,19^

### Definitions

Based on modifications of those suggested by Friedman et al.,^20^ a positive blood culture was classified as community-acquired if collected within 48 h of admission in a patient who had not been admitted to hospital in the previous 3 months, and as hospital-acquired if collected after 48 h of admission or if the patient had been admitted to hospital in the previous 3 months.^20^

### Statistical analysis

Data were analysed using Stata version 14.2 (Stata Corp, College Station, TX) and Microsoft Excel. Summary statistics were used to describe clinical, laboratory and epidemiological characteristics. Categorical variables were compared between groups using the chi-square test.

Significant differences between groups were defined as *p* ≤ 0.05. Receiver operating characteristic curves were generated to determine the optimum real-time PCR CT cut-off value for positivity. Diagnostic accuracy (sensitivity, specificity, positive predictive values and negative predictive values with 95% CI) was calculated using the DIAGTEST Stata module.

### Ethical considerations

The study was approved by the Human Research Ethics Committee at the University of Cape Town (HREC REF Number 729/2015).

## Results

A total of 212 signal-positive blood cultures with morphology meeting the specified inclusion criteria from 193 patients were selected. Multiple samples from a single patient were included if collected on different days or times or from different lumens of a central venous catheter.

### Culture results

Sub-culture from blood culture broth was positive for 195/212 (92%) samples, where *S. pneumoniae* was isolated from 55/212 (26%) samples (Figure 1a). Of these 55 samples, three (5%) were mixed cultures with *Listeria monocytogenes, Klebsiella pneumoniae* or a coagulase negative staphylococcus (CoNS). Other gram-positive organisms were cultured in 140/212 (66%) samples, whilst the remaining 17/212 samples (8%) were sub-culture negative.

**FIGURE 1:**
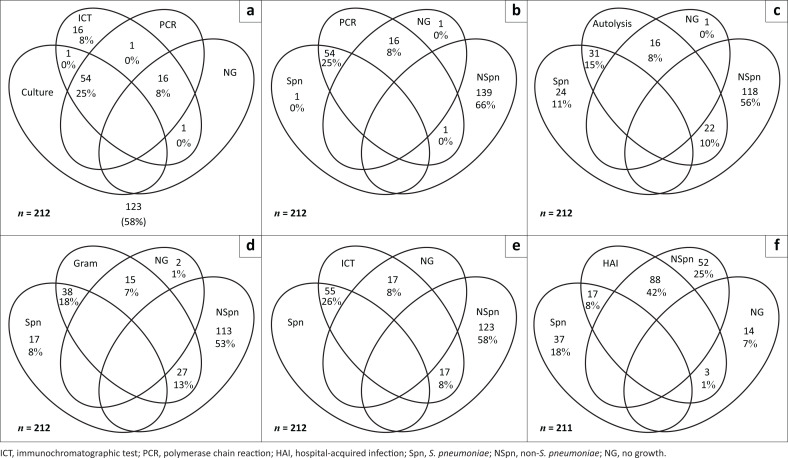
Venn diagrams representing numbers and proportions of specimens tested with (a) culture positivity versus immunochromatographic test and PCR positivity; (b) PCR positivity versus culture stratified into *Streptococcus pneumoniae*, non-*Streptococcus pneumoniae* and NG groups; (c) autolysis versus culture positivity stratified into *Streptococcus pneumoniae*, non-*Streptococcus pneumoniae* and no growth groups; (d) Gram stain with gram-positive cocci in pairs (‘Gram’) versus culture positivity stratified into *Streptococcus pneumoniae*, non-*Streptococcus pneumoniae* and no growth groups; (e) immunochromatographic test versus culture stratified into *Streptococcus pneumoniae*, non-*Streptococcus pneumoniae* and no growth groups; and (f) samples that met the study definition for hospital-acquired infection versus culture positivity stratified into *Streptococcus pneumoniae*, non-*Streptococcus pneumoniae* and no growth groups.

We stratified samples according to culture results into the *S. pneumoniae* (Spn) group, the non-*S. pneumoniae* (NSpn) (other gram-positive organisms) group and the no growth (NG) on sub-culture group. Characteristics of the groups are summarised in Table 2-A1.

Visual autolysis occurred in 33% of all samples collected (Figure 1c). The proportion of visually autolysed samples differed significantly between groups, with 94% (16/17) of samples in the NG group, 56% (31/55) of samples in the Spn group and 16% (22/140) of samples in the NSpn exhibiting visible autolysis (*p* ≤ 0.001).

### Performance of polymerase chain reaction and antigen tests

Overall, we detected *lytA* in 71/212 (33%) samples using PCR, and ICT was positive in 89/212 (42%) samples (Figure 1a). There were 193/212 (91%) samples which had concordant culture, PCR and antigen test results.

The results of the 19/212 (9%) discordant samples are summarised in Table 1.

**TABLE 1:** Discordant results between the antigen test (immunochromatographic test), polymerase chain reaction and culture.

Number	Culture result	Antigen (ICT)	PCR	CT value‡
**Culture and antigen positive for *S. pneumoniae* but PCR negative**				
1	*Streptococcus pneumoniae* + coagulase negative staphylococcus	Positive	Negative	26.00
**Culture negative for *S. pneumoniae* but antigen and PCR positive for *S. pneumoniae***				
2	Coagulase negative staphylococcus	Positive	Positive	13.76
**Culture and PCR negative for *S. pneumoniae* but antigen positive**				
3	*Enterococcus faecium* + coagulase negative staphylococcus	Weak positive	Negative	-
4	*Streptococcus agalactiae*	Weak positive	Negative	-
5	*Streptococcus anginosus*	Weak positive	Negative	-
6	*Enterococcus faecalis* + coagulase negative staphylococcus	Weak positive	Negative	-
7	Viridans streptococcus	Positive	Negative	-
8	Viridans streptococcus†	Positive	Negative	25.04
9	*Streptococcus mitis* †	Positive	Negative	26.49
10	*Streptococcus mitis* †	Positive	Negative	26.32
11	*Streptococcus mitis*	Positive	Negative	25.69
12	*Streptococcus mitis*	Positive	Negative	-
13	*Streptococcus mitis* + coagulase negative staphylococcus	Positive	Negative	-
14	*Streptococcus alactolyticus*	Positive	Negative	-
15	Coagulase negative staphylococcus	Positive	Negative	-
16	Skin flora§	Positive	Negative	29.22
17	Skin flora§	Positive	Negative	-
18	Skin flora§	Positive	Negative	-
**No growth on culture and PCR negative but antigen positive**				
19	No growth	Positive	Negative	-

ICT, immunochromatographic test; PCR, polymerase chain reaction; CT, cycle threshold; *S. pneumoniae, Streptococcus pneumoniae.*

† , Isolates from the same patient;

‡ , A cycle threshold cut-off value of ≤ 22 was used to determine PCR positivity;

§ , Skin flora were mixed viridans streptococci ± coagulase negative staphylococcus.

**TABLE 2:** Diagnostic accuracy of immunochromatographic test and Gram stain with gram-positive cocci in pairs when compared with culture, polymerase chain reaction and the composite of culture or polymerase chain reaction positivity for Streptococcus pneumoniae.

Variable	Compared to culture positivity								Compared to PCR positivity								Compared with culture or PCR positivity							
	Sensitivity		Specificity		Positive predictive value		Negative predictive value		Sensitivity		Specificity		Positive predictive value		Negative predictive value		Sensitivity		Specificity		Positive predictive value		Negative predictive value	

	%	95% CI	%	95% CI	%	95% CI	%	95% CI	%	95% CI	%	95% CI	%	95% CI	%	95% CI	%	95% CI	%	95% CI	%	95% CI	%	95% CI
**Immunochromatographic test**																								
All isolates (*n* = 212)	100	100–100	88	83–92	76	70–82	100	100–100	100	100–100	87	83–92	80	74–85	100	100–100	100	100–100	88	83–92	81	76–86	100	100–100
Gram positive cocci in pairs (*n* = 80)	100	100–100	96	92–100	97	94–100	100	100–100	100	100–100	96	92–100	98	95–100	100	100–100	100	100–100	96	92–100	98	95–100	100	100–100
History compatible with respiratory illness or meningitis (*n* = 78)	100	100–100	96	92–100	98	94–100	100	100–100	100	100–100	93	92–100	96	92–100	100	100–100	100	100–100	96	92–100	98	95–100	100	100–100
**Gram stain showing gram-positive cocci in pairs**																								
All isolates (*n* = 212)	69	63–76	81	75–86	58	52–65	87	82–92	75	69–81	81	76–86	66	60–73	86	82–91	74	68–80	81	75–86	66	60–73	86	81–90
Clinical history compatible with respiratory illness or meningitis (*n* = 78)	66	55–77	81	71–90	84	76–93	60	48–72	71	60–81	81	73–90	88	81–95	59	49–70	69	59–79	81	72–90	88	81–95	57	46–68

PCR, polymerase chain reaction; CI, confidence interval.

### Clinical, laboratory and epidemiological characteristics

Respiratory illness and meningitis predominated in the Spn (75%) and NG (65%) groups, whilst other illnesses predominated in the NSpn (61%) group and this was significant (*p* ≤ 0.001).

Gram-positive cocci in pairs were significant in the both of the Spn (69%) and NG groups (88%), whereas the proportion with this morphology in the NSpn group was only 19% (*p* < 0.001) (Figure 1d).

Most samples in the Spn (78%) and NG (88%) groups were submitted from primary/secondary level care hospitals and most samples from the Spn (67%) and NG groups (82%) fulfilled our definition for community-acquired infection. Fifty-three per cent of samples in the NSpn group were also sent from primary/secondary level care hospitals, and 63% of samples in the NSpn group fulfilled our definition for (HAI) (*p* ≤ 0.001) (Figure 1f). One sample could not be assigned to community or HAI categories because of missing data.

### Diagnostic accuracy

The sensitivity of ICT compared with culture, PCR and the composite of culture or PCR was 100%. Specificity was 88%, 87% and 88%, respectively. Positive predictive value was 76%, 80% and 81%, respectively, whilst the negative predictive value was 100%. Specificity and positive predictive value against each of these reference standards increased to 93% – 96% and 96% – 98% when applied to subsets of samples with gram-positive cocci in pairs on Gram stain, or subsets of samples with a clinical history suggestive of respiratory illness or meningitis. The immunochromatographic test was positive for all 72 samples that were positive for *S. pneumoniae* using our composite reference, even though only 53 of these samples had gram-positive cocci in pairs on Gram stain. Analysis after exclusion of duplicate samples did not alter the results significantly.

The sensitivity of classical pneumococcal Gram stain with gram-positive cocci in pairs in our study was 69%, 75% and 74% when compared with culture, PCR and the composite reference, and specificity was 81% for all three. The positive predictive values were 58%, 66% and 66%. The negative predictive values were 87%, 86% and 86%. The sensitivity decreased and specificity was unchanged when applied to the subset with a history compatible with respiratory illness or meningitis. Whilst positive predictive values increased when applied only to this subset, this was still below 90% and also resulted in a decrease in the negative predictive value of ≤ 60%. Analysis did not change significantly after duplicate samples were excluded.

## Discussion

Rapid diagnosis of IPD is challenging and current methods are limited by various factors. We aimed to demonstrate the utility of a selected ICT to rapidly identify *S. pneumoniae* on broth from selected signal-positive blood cultures.

The immunochromatographic tests were positive in all samples in the Spn (sub-culture positive for *S. pneumoniae; n* = 55) group, giving a sensitivity of 100%. Only one sample (Case 1) in this group was ICT positive but PCR negative, with a CT value of 26. Culture results of this sample indicated mixed growth of *S. pneumoniae* and a CoNS, and the elevated CT value was likely because of growth competition with the CoNS for nutrients in the blood culture broth.

The immunochromatographic tests were positive in all samples in the NG (no growth on culture; *n* = 17) group. All but one sample (1/17) in the NG group (Case 19) was PCR positive, suggesting a probable false-positive ICT result. Retesting of Case 19 with a different PCR was negative (data not shown) and the patient was subsequently diagnosed with enterovirus meningitis on viral PCR. The similarities between ICT results obtained for the Spn and NG groups support the assumption that samples in the NG group most likely represented autolysed, non-viable *S. pneumoniae* and that the addition of a culture-independent assay for these types of results may assist diagnosis.

The NSpn group (sub-culture culture positive for organisms other than *S. pneumoniae*; *n* = 140) had 17 samples which were ICT positive; of these, only Case 2 was also PCR positive (CT = 13.76). Gram-positive cocci were observed for this sample, there was no visible autolysis and culture yielded a CoNS. It is unlikely that both ICT and PCR produced false-positive results, especially with the low CT value obtained. A likely explanation is that *S. pneumoniae* was either non-viable or outcompeted by the CoNS on culture. Of the remaining ICT positive samples, 5/16 produced a detectable signal on PCR, above the determined cut-off, with CT values ranging from 25 to 29 (cases 8–11 and 16). It is possible that non-viable *S. pneumoniae* was present or outcompeted by growth of other organisms including viridans streptococci in these samples. Alternatively, the signal detected could have been because of the cross-reactivity of the PCR with other gram-positive organisms, which has mostly been described in the presence of other streptococcal species owing to their genetic relatedness.^21^

Our reported ICT sensitivity (100%) was similar to that reported by Altun and colleagues (100%) and Moisi and colleagues (87% – 100%). However, our reported specificity (87% – 88%) was higher than that reported by Altun and colleagues (64%) and lower than that reported by Moisi and colleagues (96% – 100%). Altun and colleagues compared ICT with culture, directly on signal-positive blood cultures with gram-positive cocci in pairs or chains. However, the distribution of gram-positive cocci in pairs versus chains was not described and no confirmatory molecular testing was performed; therefore, the presence of non-viable *S. pneumoniae* in samples could not be excluded.^13^ Moisi and colleagues performed ICT and *lytA* PCR directly on any signal-positive blood cultures in Mali and Thailand, irrespective of Gram stain results, and ICT was only compared with culture and not PCR as a reference method. The inclusion of any signal-positive sample, irrespective of Gram stain finding, may account for the higher value reported for specificity.^12^

Classic Gram stain morphology of *S. pneumoniae* (gram-positive cocci in pairs) performed poorly in the diagnosis of IPD, with limited sensitivity (69% – 75%), specificity (81%), positive predictive (58% – 66%) and negative predictive values (86% – 87%). Even though the positive predictive value improved to 84% – 88% when applied to the subset of samples with a history compatible with respiratory illness or meningitis, this resulted in a decrease in the negative predictive value (57% – 60%) and sensitivity (66% – 71%), limiting its utility as a standalone diagnostic tool for IPD. Specificity (81%) remained unchanged when applied to this subset.

An important limiting factor when assessing diagnostic tools for *S. pneumoniae* diagnosis is the lack of a reliable gold standard, with poor sensitivity for culture. We included *lytA* PCR as a reference standard, but used a stringent CT cut-off value to compensate for its recognised limited specificity. The CT cut-off value of ≤ 22 to determine positivity was, therefore, considerably lower than the conventional diagnostic PCR CT cut-off value (CT~37), but because the potential target organism was already enriched in the blood culture broth, and the extracted DNA diluted 1:10 prior to PCR, a lower CT cut-off value was deemed acceptable. This experimentally determined cut-off, however, resulted in one false-negative PCR result when compared with culture, and five samples, which were ICT positive and produced a signal on PCR above the determined cut-off, but were Spn culture-negative. Experimentally determined cut-off values should be used with caution and interpreted in conjunction with other laboratory findings.

We reported faint ICT results as positive, according to the manufacturer’s instructions. We noted four such weak positive results, all of which cultured other gram-positive organisms, and were PCR negative (no signal detected). Whilst the numbers were small, we recommend reporting weak positive results as negative or indeterminate.

We observed significant differences in basic clinical and epidemiological characteristics and Gram stain morphology between the Spn, NG and NSpn groups, with the majority of isolates from the Spn and NG groups exhibiting gram-positive cocci in pairs (69% and 88%), autolysis (56% and 94%) and occurring in patients with primarily community-acquired infection (67% and 82%), which were predominantly respiratory illness and meningitis (75% and 65%).

In contrast, isolates from the NSpn group exhibited gram-positive cocci in pairs in only 19% of samples, were rarely visibly autolysed (16%), were predominantly isolated from patients who met our definition for HAI (63%) and most (61%) presented with illnesses other than respiratory illness or meningitis.

There were insufficient data regarding prior antibiotic exposure to permit analysis for the different groups.

## Conclusion

We found that ICT had a high sensitivity and would therefore be a useful rule-out test for *S. pneumoniae* bacteraemia. A positive result could provide a rapid presumptive diagnosis of *S. pneumoniae* bacteraemia, especially where Gram stain morphology and clinical history are available to offset limitations in specificity. We conclude that the BinaxNOW pneumococcal antigen test is a useful adjunct for the diagnosis of pneumococcal bacteraemia. The added cost of the BinaxNOW pneumococcal antigen test from Alere was modest compared with DNA extraction and PCR. In our setting, where high-level penicillin resistance is relatively rare, the early identification of *S. pneumoniae* permits rapid de-escalation to penicillin in the appropriate non-meningitis clinical setting, with the potential to impact patient care and improve antibiotic stewardship. The immunochromatographic test is potentially a suitable alternative to PCR for the detection of *S. pneumoniae* in culture-negative specimens, but because of small numbers of culture-negative samples in our study, this should be further validated.

## Figures and Tables

**FIGURE 1-A1:**
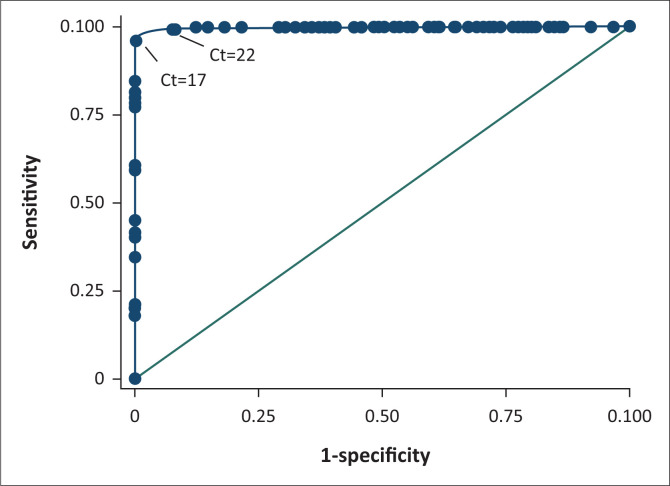
Receiver operating characteristic (ROC) curve analysis was used to determine the optimal CT cut-off value for determining a positive PCR result. Samples culturing S. *pneumoniae* were regarded as true positives, and samples culturing organisms other than S. *pneumoniae* were regarded as true negatives for this analysis. ROC curve analysis suggested that the optimal CT cut-off value for determining a positive PCR result was between 17 and 22 (AUC 0.99), and the upper value of 22 was selected.

**FIGURE 2-A1:**
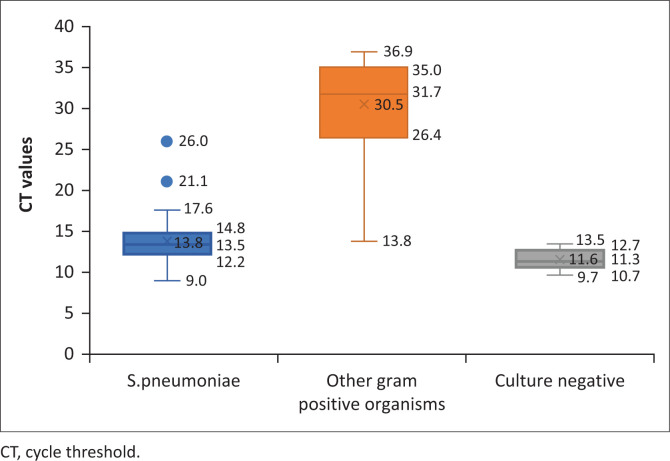
Distribution of PCR cycle threshold (CT) values using box and whisker diagrams, in signal-positive blood culture samples that have been grouped based on culture results. Whiskers represent the minimum and maximum values of the CT-values and boxes represents the interquartile range. ‘X’ represents the mean of CT-values. For the S. *pneumoniae* group, there were 2 CT-values that were outliers (excluded from box and whisker diagram) and these are represented by dots in the figure.

## Appendix 1

**TABLE 1-A1:** Primer and probe sequences used for *lytA* PCR assay.

Primer or probe	Sequence
SPN Forward primer	5’- TCG TGC GTT TTA ATT CCA GCT -3’
SPN Reverse primer	5’– ACG CAA TCT AGC AGA TGA AGC A –3’
SPN Probe	5’- /56-FAM/CTC CCT GTA /ZEN/TCA AGC GTT TTC GGC A/3IABkFQ/ -3’

**TABLE 2-A1:** Clinical, laboratory and epidemiological characteristics of samples grouped according to culture.

Variable	Group						Statistical comparison (*p*-value)		

	Spn (*N* = 55)		NSpn (*N* = 140)		NG (*N* = 17)		Spn vs NSpn vs NG	Spn vs. NSpn	Spn vs. NG
	*N*	%	*N*	%	*N*	%			
**Clinical**	-	-	-	-	-	-	< 0.001	< 0.001	0.884
Respiratory illness	34	62	22	16	9	53	-	-	-
Meningitis	7	13	4	3	2	12	-	-	-
Other	2	4	86	61	1	6	-	-	-
No data	12	22	28	20	5	9	-	-	-
**Autolysis†**	-	-	-	-	-	-	< 0.001	< 0.001	0.004
Yes	31	56	22	16	16	94	-	-	-
No	24	44	118	84	1	6	-	-	-
**Gram stain**	-	-	-	-	-	-	< 0.001	< 0.001	0.112
Pairs	38	69	27	19	15	88	-	-	-
Chains	16	29	63	45	1	6	-	-	-
Cocci	1	2	50	36	1	6	-	-	-
**Healthcare facility of origin**	-	-	-	-	-	-	< 0.001	0.001	0.360
Tertiary level care hospital	12	22	66	47	2	12	-	-	-
Primary or secondary level care hospitals	43	78	74	53	15	88	-	-	-
**Community versus nosocomial infection** ‡	-	-	-	-	-	-	< 0.001	< 0.001	0.461
Community	37	67	52	37	14	82	-	-	-
Nosocomial	17	31	88	63	3	18	-	-	-
No data	1	2	0	0	0	0	-	-	-

Spn, Growth of *S. pneumoniae* on culture; NSpn, Growth of an organism other than *S. pneumoniae* on culture; NG, No growth of any organism on culture.

† , Autolysis was determined by the subjective presence of brown discoloration of the blood culture broth;

‡ , Community versus nosocomial infection was determined as per the study definition.
